# Targeting UDP-Galactopyranose Mutases from Eukaryotic Human Pathogens

**DOI:** 10.2174/1381612811319140007

**Published:** 2013-04

**Authors:** Karina Kizjakina, John J Tanner, Pablo Sobrado

**Affiliations:** 1 Department of Biochemistry, Virginia Tech, Blacksburg, VA 24061, USA; 2 Departments of Chemistry, University of Missouri-Columbia, Columbia, MO 65211, USA; 3 Departments of Biochemistry, University of Missouri-Columbia, Columbia, MO 65211, USA

**Keywords:** UDP-galactopyranose mutase, enzyme drug target, galactofuranose, non-redox reaction, flavoenzyme, galactopyranose, inhibitors, eukaryotic pathogens.

## Abstract

UDP-Galactopyranose mutase (UGM) is a unique flavin-dependent enzyme that catalyzes the conversion of UDP-galactopyranose (UDP-Gal*p*) to UDP-galactofuranose (UDP-Gal*f*). The product of this reaction is the precursor to Galf, a major component of the cell wall and of cell surface glycoproteins and glycolipids in many eukaryotic and prokaryotic human pathogens. The function of UGM is important in the virulence of fungi, parasites, and bacteria. Its role in virulence and its absence in humans suggest that UGM is an ideal drug target. Significant structural and mechanistic information has been accumulated on the prokaryotic UGMs; however, in the past few years the research interest has shifted to UGMs from eukaryotic human pathogens such as fungi and protozoan parasites. It has become clear that UGMs from prokaryotic and eukaryotic organisms have different structural and mechanistic features. The amino acid sequence identity between these two classes of enzymes is low, resulting in differences in oligomeric states, substrate binding, active site flexibility, and interaction with redox partners. However, the unique role of the flavin cofactor in catalysis is conserved among this enzyme family. In this review, recent findings on eukaryotic UGMs are discussed and presented in comparison with prokaryotic UGMs.

## 1. INTRODUCTION

Vector-borne diseases like Chagas disease and leishmaniasis are caused by parasitic human pathogens and are a major health burden in many developing countries. Current therapies are not very effective and suffer from toxic side effects [1-2]. In addition, the emergence of drug-resistant strains has been reported [3-6]. These vector-borne diseases have been recognized by the World Health Organization (WHO) as Neglected Tropical Diseases (NTD) – chronic infectious diseases endemic mainly in underdeveloped countries, and even though millions of people are affected and thousands die every year, there are no effective cures [7]. In recent years, significant research efforts have been focused on NTD due to policies and research programs implemented by the WHO and other governmental and private organizations [8]. Fungi from *Aspergillus *species cause a series of broncho-respiratory infections collectively known as aspergillosis [9-10]. Infections by *Aspergillus fumigatus* are the most common in immuno-compromised individuals. Once infection has been established, the mortality rate can be close to 50% [11]. Therefore, new effective anti-fungal drugs are urgently needed.

A possible mode of intervention against these parasitic and fungal pathogens is to inhibit the activity of enzymes that aid in cell wall biosynthesis and/or host-pathogen interactions [12-13]. It has recently been shown that galactofuranose (Gal*f*), a sugar not found in humans, plays an important role in cell wall biosynthesis in *A. fumigatus* and many bacteria and is a major component of the cell surface matrix of *Trypanosoma cruzi* and *Leishmania major*, the causative agents of Chagas disease and leishmaniasis, respectively. In these parasites, Gal*f* plays a major role in virulence [14]. The flavoenzyme UDP-galactopyranose mutase (UGM) is a unique enzyme not present in humans and is essential in the biosynthesis of Gal*f*. Here, we provide an overview of the biosynthesis of Gal*f* and its role in pathogenesis in eukaryotic pathogens with a focus on recent studies on eukaryotic UGMs from *T. cruzi*, *L. major*, and *A. fumigatus*.

### 1.1 Neglected Tropical Diseases Caused by* T. cruzi *and* L. major*

Chagas disease (or American trypanosomiasis) is endemic throughout Central and South America. It is caused by the protozoan parasite *T. cruzi* and is usually transmitted through a sylvatic cycle from an infected triatomine (“kissing bug”) vector that lays parasite-laden feces on wounds and mucous membranes, as well as in conjunctivas [15]. In addition, there have been reports of infection via blood transfusion and orally through ingesting infected mother’s milk, raw and undercooked meat, or other food infected by triatomines and/or their feces [16]. Very often in its early stages, Chagas disease is asymptomatic. If left untreated, parasite invasion becomes a serious health risk; symptoms can develop 10-20 years later when the disease becomes chronic and has high mortality rates, typically due to the parasitosis of the heart, causing myocarditis [17]. It is estimated that approximately 16-18 million people have Chagas disease and approximately 50,000 of them die annually; however, these numbers could be higher, since infections are often misdiagnosed due to the very limited, or sometimes complete lack of symptoms [15, 18]. 

Leishmaniasis is a vector-borne systemic disease caused by a trypanosomatid protozoa from the *Leishmania spp.*, which invade human macrophages and replicate intracellularly after being transmitted to humans by infected sandflies (genera *Phlebotomus* and *Lutzomyia*) [19]. Depending on the particular parasitic species, leishmaniasis can develop into three forms: cutaneous, mucocutaneous, or visceral leishmaniasis [20]. *L. major* is the causative agent of cutaneous leishmaniasis, which manifests as a severe skin infection that often causes disfigurement and is endemic in developing countries in the tropics, subtropics, and the Mediterranean basin, with thousands reported new cases annually [7].

### 1.2 Infections Caused by *A. fumigatus*

Fungi of the genus *Aspergillus* are responsible for several human diseases ranging from allergic reactions and lung infections to sepsis and death [9]. There are hundreds of members of the *Aspergillus *genus,**and some are pathogenic to humans, with *A. fumigatus *and* A. niger *being the most common [21-23]. Among the diseases related to *Aspergillus* infection, allergic bronchopulmonary aspergillosis (ABPA) and invasive pulmonary aspergillosis (IPA) represent a significant health threat to both immuno-competent and immuno-compromised persons [9, 24]. IPA infections are commonly observed in patients receiving chemotherapy, organ transplants, and in late-stage AIDS [25-26]. An increase from 0.3% to 5.8% in IPA infections in patients admitted to intensive care units (ICUs) has been reported in recent years, and has been accompanied by a high mortality rate (50-70%) [27-28]. This demonstrates the need for new anti-fungal drugs to combat *Aspergillus* infections.

## 2. ROLE OF GALACTOFURANOSE IN VIRULENCE

**Fig. (1):**
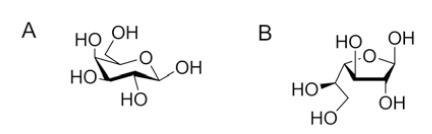
Structures of *β*-D-galactopyranose (**A**) and *β*-D-galactofuranose (**B**).

Galactose is a hexose and a C-4 epimer of glucose (Fig. **1**). In mammals, galactose exists only in the pyranoside form (Gal*p*) linked to other carbohydrates as an essential component of glycolipids and glycoproteins [29]. The main source of galactose in humans comes from consumption of dairy products and its metabolism occurs through the Leloir or Isselbacher pathways [30-31]. Galactose in the furanoside (Gal*f*) form is not found in mammals; however, Gal*f* is an important building block of glycans of the cell wall and cell surface in several pathogenic organisms and, therefore, its biosynthesis is a strategic target in the discovery of anti-microbial treatments [14]. The specific role of Gal*f*-containing molecules in *Leishmania spp.*, *T. cruzi*, and *A. fumigatus* is described in this section.

### 2.1 Galactofuranose in *T. cruzi*

In *T. cruzi*, *β*-Gal*f* is found in glycoinositolphospholipids (GIPLs) and glycosylphosphatidylinositol (GPI) anchor proteins [32-33]. These glycoconjugates are highly expressed throughout the life cycle of *T. cruzi* and are the main component of the parasite dense surface coat, which has a protective function in parasite survival in the hydrolytic and digestive environment inside their hosts and are important for proliferation [34-36]. For instance, a 45 kDa GPI-mucin is expressed only in invasive trypomastigotes and not in non-invasive amastigotes [37]. Using specific monoclonal antibodies against this protein prevented adhesion of *T. cruzi* to heart myoblasts [37]. These results suggest that Gal*f*-containing glycoconjugates are involved in the mechanism of myocardial invasion by *T. cruzi.*

### 2.2 Galactofuranose in *Leishmania spp*

In *L. major*, Gal*f* is found in the oligosaccharide core of lipophosphoglycans (LPG) and glycoinositolphospholipids (GIPL) that are essential for parasite survival in the midgut of the vector insect and for parasite transmission to the mammalian host [38-40]. GIPL-1 from *L. major* has been shown to contribute to the infection process [41-42]. LPG deletion mutants in *L. major* showed LPG involvement in resistance to oxidative stress and evasion of the human immune system [39-40].

### 2.3 Galactofuranose in *A. fumigatus*

Of the vast *Aspergillus *genus that includes over 185 species,* A. fumigatus* and *A. niger* are among the ~20 reported human fungal pathogens that cause a variety of opportunistic diseases facilitated by the suppression of the immune system [43]. Gal*f* has been identified in both organisms and is an important component in the fungal cell wall assembly, where it was found in galactomannan, glycoproteins, sphingolipids, and lipid-linked glycans [44-48]. In *A. fumigatus*, Gal*f* accounts for up to 5% of the dry weight, and is important for fungal growth and cell wall biosynthesis, cell morphogenesis and wall architecture, hyphal adhesion, spore development, and pathogenesis [22-23, 49].

## 3. UDP-GALACTOPYRANOSE MUTASE: AN ATTRACTIVE DRUG TARGET AGAINST EUKARYOTIC HUMAN PATHOGENS

**Fig. (2):**
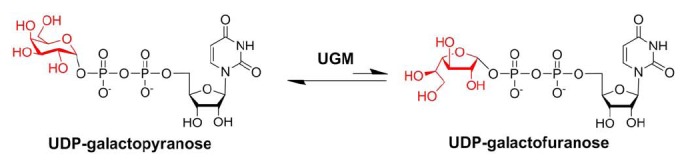
Reaction catalyzed by UDP-galactopyranose mutase.

UDP-Galactopyranose mutase (UGM) is a flavin-dependent enzyme that catalyses the isomerization of UDP-Gal*p* to UDP-Gal*f* through a unique type of flavin-dependent catalysis (Fig. **2**) [13, 50-52]. The gene encoding for UGM (*glf*) was first identified in prokaryotes in 1996 while studying the* Escherichia coli* K12 O antigen [53]. In the following years, it was identified in other pathogens including the eukaryotes *L. major*, *T. cruzi*, and *A. fumigatus* [54]. Deletion of the UGM gene leads to attenuated virulence in *L. major* [55]. In *T. cruzi*, the role of Gal*f* in binding to mammalian cells has been shown; however, deletion of the UGM gene in this parasite have not been performed.

Deletion of the UGM gene in *A. fumigatus*, in addition to causing attenuated virulence, leads to cell-wall morphology defects, increased sensitivity to anti-fungal drugs, and growth reduction [21-22]. These results validate UGM as a potential target for the development of drugs against these eukaryotic pathogens.

### 3.1 Primary Structure of UGMs

**Fig. (3):**
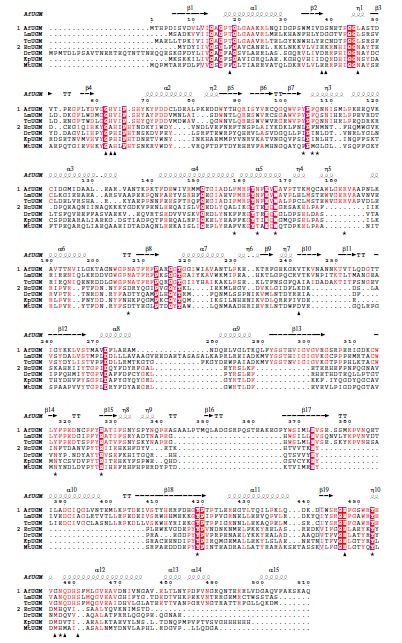
Sequence alignment of eukaryotic (group 1) and prokaryotic (group 2) UGMs. The residues conserved in all of the sequences are shown in red shaded boxes. Those conserved only in one group are shown in red color. Active site residues are marked with a star, and those interacting with flavin are marked with triangles. The α-helix (spiral) and β-sheets (arrows; TT – strict β-turns) of *Af*UGM are depicted on top. ClustalW was used to generate the alignment and ESPript 2.2 to create the figure. (The color version of the figure is available in the electronic copy of the article).

The polypeptide chain lengths of eukaryotic UGMs are generally about 100 amino acid residues longer than those of the prokaryotic enzymes (Fig. **3**). Sequence alignment reveals a moderate identity (47-60%) among the eukaryotic UGMs from *A. fumigatus* (*Af*UGM), *L. major* (*Lm*UGM), and *T. cruzi* (*Tc*UGM), and a slightly lower (37-44%) sequence identity among the prokaryotic homologs from *Escherichia coli* (*Ec*UGM), *Mycobacterium tuberculosis* (*Mt*UGM), *Klebsiella pneumoniae* (*Kp*UGM), and *Deinococcus radiodurans* (*Dr*UGM). However, the sequence identity between eukaryotic and prokaryotic UGM groups is surprisingly low (14-18%) (Tab. **1**). Conserved among all UGMs is the GxGxxG motif that is necessary for FAD binding. Only partial conservation of active site residues is observed (Fig. **3**). The low amino acid conservation and the extra amino acid sequence in eukaryotic UGMs endows these enzymes with unique structural features that are important for enzyme function; these are discussed in the next section. Interestingly, an obvious NAD(P)H binding domain or motif is not found in this family of enzymes. This is intriguing since this class of enzymes has been shown to function only in the reduced state.

### 3.2 3-Dimensional Structure of UGMs

**Fig. (4):**
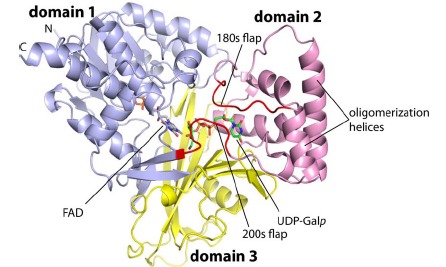
Protomer structure of reduced *Af*UGM complexed with UDP-Gal*p*. FADH^-^ and UDP-Gal*p* are colored gray and green, respectively. The flexible active site flaps are colored red. (The color version of the figure is available in the electronic copy of the article).

Whereas several crystal structures of bacterial UGMs have been determined [56-58], among the eukaryotic enzymes, only the structure of *Af*UGM is known at this time (Tab. **2**) [59-60]. *Af*UGM is a mixed α/β fold protein containing three structural domains (Fig. **4**). Domain 1 includes a Rossmann fold core and participates in FAD binding. Domains 2 and 3 function in substrate binding [59]. This general 3-domain architecture is also found in the bacterial enzymes; however, the eukaryotic enzymes have extra structural elements that are important in oligomerization and substrate recognition, as summarized below.

The conformations of the flavin and flavin-protein interactions are highly conserved between prokaryotic and eukaryotic UGMs (Tab. **3**). The isoalloxazine ring of the oxidized enzyme is planar, which is typical for flavoenzymes. Characterization of the reduced FAD conformation is important for understanding the chemical mechanism because the reduced FAD functions as a nucleophile in the UGM reaction. This function places certain structural restrictions on the flavin isoalloxazine. In particular, steric considerations suggest that the reduced isoalloxazine should be nonplanar with the wings of the isoalloxazine bending away from the substrate. Indeed such a conformation is observed in reduced *Af*UGM and *Dr*UGM. In both cases, the isoalloxazine exhibits a butterfly-like deviation from planarity in which the pyrimidine ring bends ~7º away from the substrate site such that the *si* face is concave [56, 59]. Curiously, bending of the isoalloxazine by ~13º in the opposite direction is observed in reduced *Kp*UGM; the relevance of this conformation is uncertain since it appears to be inconsistent with nucleophilic attack [61-62].

**Fig. (5):**
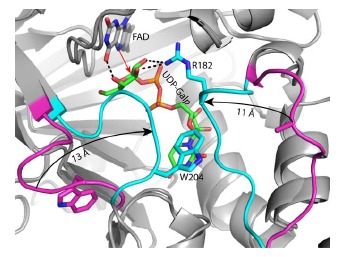
Close-up view of the active site of reduced *Af*UGM complexed with UDP-Gal*p* highlighting flap closure. The flaps of the ligand-free reduced enzyme are colored magenta, while those of the UDP-Gal*p* complex are colored cyan. The black arrows denote the direction of flap closure induced upon substrate binding. The red arrow denotes the direction of nucleophilic attack by the flavin N5 on the anomeric C atom of the substrate. (The color version of the figure is available in the electronic copy of the article).

Comparison of the structures of *Af*UGM and bacterial UGMs complexed with UDP-Gal*p* reveals conserved motifs and important differences. In all the complex structures (Tab. **2** and Tab. **3**), the OH-4 of the Gal*p* moiety interacts with the flavin O-4 through hydrogen bonding, and the anomeric carbon of the sugar is placed within a short distance from the flavin N-5 (Fig. **5**). Also, several Arg and Tyr residues are conserved and participate in electrostatic interactions with the pyrophosphate portion of UDP-Gal*p* (Tab. **3**). In contrast to bacterial UGMs, in *Af*UGM the OH-6 of Gal*p* is rotated by 110º. There is also a variation in the conformation of bound UDP. In *Af*UGM, UDP is displaced by ~4 Å and rotated by about 90º with respect to bacterial *Kp*UGM and *Dr*UGM. This allows for the hydrogen bonding of uracil with Gln107, a residue that is not present in bacterial UGMs. These structural differences in substrate recognition between bacterial and eukaryotic UGMs could have implications for inhibitor discovery. In particular, it seems unlikely that compounds that target the uridine site of bacterial UGMs will be effective against eukaryotic UGMs. 

Large protein conformational changes (>10 Å movements) accompany substrate binding in UGMs. *Af*UGM, and presumably other eukaryotic UGMs, exhibit larger conformational changes. Comparison of the structures of the substrate-free and substrate-bound forms revealed two flaps (residues 179–187 and 203–209) that close down over the substrate like the flaps of a box top (Fig. **5**). Whereas the 180s flap is analogous to the mobile loop of bacterial UGMs, the 200s flap is unique to eukaryotic UGMs. The dramatic closing of the active site in bacterial and eukaryotic UGMs is an important aspect of the catalytic mechanism. These movements result in the assembly of the constellation of residues that position the substrate for nucleophilic attack by the FAD. Furthermore, the closing of the active site prevents diffusion of intermediates, such as UDP, out of the active site during the catalytic cycle.

**Fig. (6):**
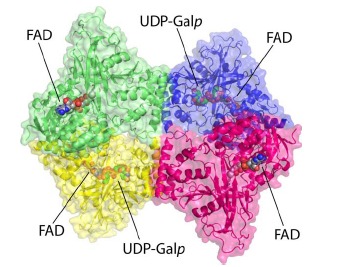
Structure of the *Af*UGM tetramer. Each protomer has a different color. The yellow protomer has the same orientation as the protomer in Fig. **4**. (The color version of the figure is available in the electronic copy of the article).

Various oligomeric states have been observed for UGMs in solution. The oligomeric states of several UGMs have been determined from size exclusion chromatography, small-angle X-ray scattering (SAXS), and analysis of protein-protein interfaces in crystal lattices (Tab. **4**). Bacterial UGMs tend to form dimers in solution. *Ec*UGM, *Kp*UGM, and *Mt*UGM form a semicircular dimer [58, 63]. The fact that this structure is formed by multiple UGMs in different crystal lattices attests to its veracity. The oligomeric state of *Dr*UGM is less certain. The classic UGM semicircular dimer is not found in the *Dr*UGM lattice, and solution studies of the oligomeric state have not been performed on the enzyme. The *Dr*UGM crystal lattice implies decameric and dimeric assemblies, but clearly additional work is needed to determine the oligomeric state and quaternary structure of *Dr*UGM. In contrast, the oligomeric state and quaternary structure of *Af*UGM have been unequivocally determined using a combination of SAXS and X-ray crystallography [59]. These studies have shown that *Af*UGM is unique among UGMs in that it forms a tetramer in solution [59, 64]. The *Af*UGM tetramer is a dimer-of-dimers assembly (Fig. **6**). Unique structural features of *Af*UGM that are absent in the bacterial enzymes enable tetramerization. These extra elements include a longer C-terminus, an extra helix in domain 2, and extension of another helix of domain 2 (Fig. **4**).

Because the reduced FAD is essential for catalysis, the mechanism by which the enzyme is activated by flavin reduction is an important aspect of UGM biochemistry. Insight into the structural underpinnings of this mechanism has been obtained by comparing crystal structures of oxidized and reduced UGMs. Inspection of the bacterial enzyme structures reveals little difference between the oxidized and reduced conformations, aside from the bending of the isoalloxazine described above.

**Fig. (7):**
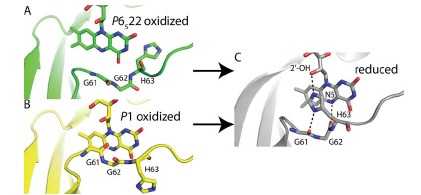
Conformations of the histidine loop in *Af*UGM structures. **A**, oxidized *P*6_5_22 form (PDB code 3UTE); **B**, oxidized P1 form (PDB code 3UKH); **C**, reduced enzyme (PDB code 3UTF).

Initial results for *Af*UGM potentially reveal a much more complex mechanism for activating eukaryotic UGMs (Fig. **7**). Two crystal forms of *Af*UGM have been described, a *P*6_5_22 form reported by us [59], and a *P*1 form reported by Sanders’ group [60]. Although the interpretation of these structures is complicated by the binding of sulfate ion in the* P*6_5_22 form, and weak electron density in the *P*1 form, the structures tantalizingly imply large conformational changes involving the conserved histidine loop (G61-G62-H63). The structures show that, in the oxidized enzyme, conserved His63 is near the pyrimidine ring of the isoalloxazine and the carbonyl of Gly62 points away from the isoalloxazine (Fig. **7A** and **7B**), which is unprecedented for UGMs. The structures further indicate that flavin reduction induces a crankshaft rotation of the loop backbone, which reverses the orientation of the Gly62 carbonyl bond vector and moves the imidazole of His63 by over 5 Å. These changes bring the carbonyl of Gly62 within hydrogen bonding distance of the N5 of the reduced flavin and move His63 to the *si* face of the isoalloxazine where it stacks in parallel with the isoalloxazine and forms a hydrogen bond with the OH-2’ of the ribityl (Fig. **7C**). These interactions between the histidine loop and the flavin help stabilize the reduced state of the enzyme and are found in all other UGM structures. The presence of two Gly residues in the loop is unique to eukaryotic UGMs and probably accounts for the large conformational changes seen in *Af*UGM compared to the bacterial enzymes. More research is needed to validate these conformational changes for *Af*UGM and determine whether other eukaryotic UGMs exhibit analogous movements.

### 3.3 Chemical Mechanism of Eukaryotic UGMs

Despite structural differences, the unique chemical mechanism utilized by UGMs is conserved among the members of this enzyme family. For all UGMs, only the reduced form of the enzyme is active [63], although the reaction does not involve a net gain or loss of electrons, which is common among many other classes of FAD-dependent enzymes [65-67]. The reported steady-state values with UDP-Gal*f* as substrate and dithionite as the reductant show only minor differences in *k_cat_*and *k_cat/_K_M_* among members of the UGM family (Table **5**).

**Fig. (8):**
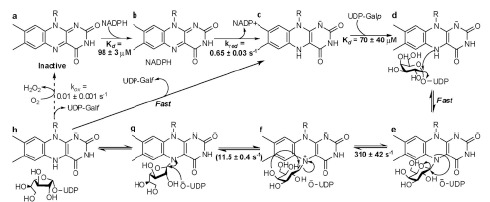
Proposed mechanism for *Tc*UGM. The oxidized enzyme binds and reacts with NADPH. UDP-Gal*p* binds to the reduced enzyme and a flavin-sugar adduct is formed rapidly by the direct attack of the flavin. Formation of the flavin iminium ion leads to opening of the sugar ring. Attack of the UDP to form the UDP-Gal*f* and its release occur rapidly. The reaction can occur for several more cycles (~1000) before the enzyme is oxidized by molecular oxygen. The rate limiting step is proposed to be the closing of the sugar ring [73].

The enzymatic reaction has been shown to involve cleavage of an anomeric bond and the formation of a Gal*p*-FAD adduct (Fig. **8**) [68]. This process was initially thought to involve one of three mechanisms: a single-electron transfer from the reduced flavin to a postulated oxocarbenium intermediate of Gal*p* [69-70] or a nucleophilic substitution via an S_N_1 or S_N_2 mechanism, both leading to the formation of a Gal*p*-FAD adduct [71-72]. While the Gal*p* is bound to the FAD it undergoes ring opening and closing rearrangement and, after nucleophilic attack by UDP^-^, the UDP-Gal*f* is produced. Formation of the FAD-sugar adduct has been demonstrated by chemical quenching, trapping, and characterization by mass spectrometry in both eukaryotic and prokaryotic UGMs [71, 73]. Rapid reaction kinetic analysis with reduced *Tc*UGM and UDP-Gal*f* did not show the presence of a transient flavin semiquinone, inconsistent with a single electron transfer step. Instead, absorbance changes consistent with the formation of a flavin iminium ion, were observed and occur very fast [73]. The structures of *Af*UGM and prokaryotic UGMs in complex with UDP-Gal*p* clearly show that the substrate binds in a conformation optimal for direct attack by the flavin N5. Furthermore, linear free energy relationship (LFER) studies with prokaryotic UGM, reconstituted with various FAD analogs, show changes in *k_cat_* values that correlate linearly with changes in the nucleophilicity of the flavin N5 (slope of ρ = –2.4 ± 0.4), which is consistent with an S_N_2 mechanism [72]. Viscosity effect studies showed that product release was not rate limiting in the case of *Tc*UGM [73].

* In vivo*, all UGMs function in an aerobic environment. Therefore, a system for the generation and maintenance of the reduced flavin must exist in the cell. Despite not having found a canonical NAD(P)H binding motif in the primary sequence of eukaryotic UGMs, NAD(P)H was identified as an effective electron donor for the reduction of the flavin cofactor in *Tc*UGM [73]. Kinetic analyses show that there is preference for NADPH, as it reduces the flavin 7 times faster and binds 5 times tighter than NADH (Tab. **6**). In contrast, *Mt*UGM is unable to effectively react with reduced coenzymes [73]. It has been previously reported that the activity of *Kp*UGM was enhanced by the addition of NADH or NADPH [74-75]. The binding affinities or rates of reduction were not reported, however, the rate enhancement was observed at concentrations greater than 20 mM NADH and at incubation times longer than 10 minutes [74]. Taking into account that NADPH is capable of reducing *Tc*UGM with a rate constant in the second time scale and it binds with micromolar affinity, it is clear that relative to eukaryotic UGM, the bacterial enzymes are not effective NAD(P)H oxidases.

The mechanism shown in (Fig. **8**) was recently proposed for *Tc*UGM [73]. Although, as mentioned above, the initial steps in the catalysis, NAD(P)H binding and subsequent FAD reduction, occur much less effective in prokaryotic UGMs, the steps leading to the conversion of Gal*f* are conserved in these enzymes [71, 73].

## 4. METHODS FOR HIGH THROUGHPUT SCREENING FOR UGM INHIBITORS

With the exponential advance of robotics, data collection, and analysis methods, high throughput screening (HTS) provides an effective and relatively fast preliminary analysis of chemical libraries composed of thousands of chemical compounds for the search of potential chemotherapeutics [76-80]. Whether the goal is to find an effective inhibitor for a well-explored enzyme or to match existing drugs to new macromolecular targets, HTS provides the rational starting point in the drug discovery process. Elimination of ineffective drug candidates early on using HTS is essential and saves time and resources during later stages of drug development, since libraries can contain thousands of compounds with a 0.1-0.2% probability of identifying positive hits [81-82]. Thus, the development of a successful assay for HTS is extremely important.

**Fig. (9):**
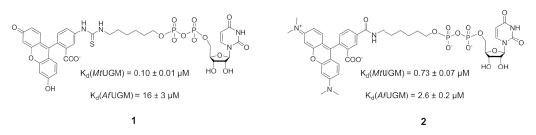
Fluorescent probes used in HTS assays for the identification of UGM inhibitors.

Standard methods used to assay UGMs include: HPLC analysis, UV/Vis and stopped-flow spectroscopy, redox potentiometry, fluorescence polarization, and radiochemical detection [64, 69, 71, 83-84]. The HPLC method has been adopted by many groups, as it easily allows one to measure the activity of UGMs both qualitatively and quantitatively. In general, the assay monitors the reverse reaction, UDP-Gal*f* to UDP-Gal*p* conversion. Both the substrate and the product are easily detected at 262 nm, which corresponds to the absorbance maxima of UDP. Despite the broad utilization of the HPLC method by many research groups, this assay is not suitable for screening large chemical libraries because of the lengthy HPLC run times and because it is not suitable for running multiple measurements at once. A radioactive assay based on the generation and monitoring of tritiated formaldehyde, from the radioactive UDP-Gal*f* degradation product, was used in the screening of 1,300 potential inhibitors against prokaryotic *Mt*UGM. However, the poor sensitivity of the assay due to the equilibrium of the reaction not favoring the formation of UDP-Gal*f* isomer was an essential drawback of this approach for high throughput screening applications [84]. Other reported assays used in HTS against UGMs are based on fluorescence polarization (FP) [83, 85-86]. FP relies on changes in the tumbling of a chromophore as it transitions from the enzyme-bound to the free-state due to competition by an inhibitor. This assay is simple, fast, and can be done on a small scale. Various fluorescent probes based on UDP were synthesized to develop a FP assay for both prokaryotic and eukaryotic UGMs (Fig. **9**). Chromophore **1** was shown to effectively bind prokaryotic UGMs from *M. tuberculosis* and *K. pneumoniae* and used in HTS with the library strategically derivatized from a thiazolidinone core [85]. Contrary to prokaryotic UGMs, the fluorescein fluorophore was not as effective with *Af*UGM, and TAMRA analog **2** was developed instead. Chromophore **2** was shown to bind to *Af*UGM with relative high affinity, thus, yielding a potential tool for HTS in search of UGM inhibitors in eukaryotes [83]. The binding of UDP-chromophore to other eukaryotic UGMs is much less effective. For instance, the K_d_ value of chromophore **2** for *Tc*UGM is >20 μM, and similar low affinity is observed for *Lm*UGM (Qi and Sobrado, unpublished results). However, since the active sites of eukaryotic UGMs are highly conserved, inhibitors of *Af*UGM might also be effective against the other eukaryotic UGM homologs.

## 5. *IN SILICO* DRUG DESIGN AND ITS APPLICATIONS TO UGM

Protein crystal structures serve as the blueprints for computer-guided molecular recognition, design, and virtual-screening of drug-like molecules and diagnostic probes [87]. There are three extensively used strategies for *in silico* drug design: *de novo* design, fragment-based drug discovery [88-89], and virtual screening [90]. The first two are very similar in their algorithms and concepts: both are based on design “from scratch”, involving the screening of small pharmacophoric chemical blocks (or fragments) within the three-dimensional active site of the target enzyme. At later stages of the experiment, these fragments are further expanded (“grown”) upon other moieties or directly joined together through a chemical bond or a linker. Virtual screening deals with the vast libraries of small chemical compounds utilizing high-throughput docking and pharmacophore-based searching algorithms and can be classified into two broad categories: ligand-based or structure-based docking and scoring [90-91].

Recently, a virtual screening using various computational tools toward the identification of inhibitors against *Ec*UGM (also *Kp*UGM and *Mt*UGM) was applied to a small-molecule library comprised of 84,000 compounds (LeadQuest,Tripos, Inc.) [57]. A total of 13 compounds (0.015% of the library) were identified as positive hits and tested for inhibitory activity toward *Kp*UGM and *Mt*UGM. Only three compounds were shown to be effective inhibitors and had comparable affinity with the best previously published prokaryotic UGM inhibitors (IC_50_ 7.2 - 62 µM, [84-86, 92-93]). The effective application of *in silico* screening to bacterial UGMs suggests that a similar approach can be applied to eukaryotic enzymes. Furthermore, it is expected that an *in silico* screening approach with the structure of *Af*UGM will also identify potential inhibitors for *Tc*UGM and *Lm*UGM.

## 6. CONCLUDING REMARKS

Gal*f* is important for cell wall biosynthesis and cell surface glycan structures in bacteria, fungi, and parasites. Gal*f* is either essential for growth or important for pathogenesis, making enzymes in its biosynthetic pathway potential drug targets. In this pathway, UGM is an ideal target for drug discovery because this enzyme is absent in humans, and its structure and chemical mechanism are unique. During the past decade, the catalytic mechanism was fully elucidated and the structural differences between prokaryotic and eukaryotic UGM characterized. Recent discoveries in the field of eukaryotic UGMs set the stage for the identification of inhibitors that might lead to drugs for the treatment of neglected diseases like Chagas disease, leishmaniasis, and fungal infections caused by *Aspergillus spp*. In principle, every conformation along the catalytic cycle is a potential design target, including both the active, reduced enzyme and the inactive, oxidized one. Strategies toward developing effective drugs can include the design of a small-molecule competitive inhibitor with much higher binding affinity to UGM with respect to UDP-Gal*p*/*f*, or even molecules that do not bind to the active site but interact with the mobile loops to prevent proper binding of the substrate.

## Figures and Tables

**Table 1.** Primary Structure Comparison of UGMs from Different Organisms.

	*Tc*UGM	*Lm*UGM	*Ec*UGM	*Kp*UGM	*Mt*UGM	*Dr*UGM
*Af*UGM	**47.0**	**49.4**	14.0	15.3	15.2	17.8
*Tc*UGM		**60.1**	16.8	18.3	14.5	15.2
*Lm*UGM			15.8	16.8	14.2	15.4
*Ec*UGM				**38.7**	**44.4**	**37.4**
*Kp*UGM					**42.1**	**39.0**
*Mt*UGM						**37.6**

Percentage Identity Shown in Bold Corresponds to the UGMs from the Same Class (Prokaryotic or Eukaryotic). ClustalW Program was Used to Calculate Percentage Identity

**Table 2.** Available UGM Crystal Structures in the Protein Data Bank (PDB)

UGM	Active site ligand	PDB code [94]	Ref.
*Af*UGM_red_		3UTF	[59]
*Af*UGM_red_	UDP	3UTG	[59]
*Af*UGM_red_	UDP-Gal*p*	3UTH	[59]
*Af*UGMox	sulfate	3UTE	[59]
*Af*UGM_red_	UDP-Gal*p*	3UKF	[60]
*Af*UGM_red/ox_	UDP-Gal*p*	3UKH	[60]
*Af*UGM_ox_	UDP	3UKL	[60]
*Ec*UGM_ox_		1I8T	[63]
*Kp*UGM_ox_	FMN	3KYB	n.a.*
*Kp*UGM_ox_	UMP, UDP-Glc*p*	3GF4	[61]
*Kp*UGM_ox_	UDP-Gal*p*	3INR	[62]
*Kp*UGM_red_	UDP, UDP-Gal*p*	3INT	[62]
*Kp*UGM_red_		1WAM	[58]
*Kp*UGM_ox_		2BI7	[58]
*Kp*UGM_red_		2BI8	[58]
*Mt*UGM_ox_		1V0J	[58]
*Dr*UGM_ox_	UDP	3HE3	[56]
*Dr*UGM_red/ox_	UDP-Gal*p*	3HDY	[56]
*Dr*UGM_ox_	UDP-Gal*p*	3HDQ	[56]
*Dr*UGM_ox_	UDP, UDP-CH_2_-Gal*p*	3MJ4	[95]

* Gruber TD, Dimond MC, Kiessling LL, Forest KT, Structure of UDP-galactopyranose mutase bound to flavin mononucleotide. Unpublished results.

**Table 3.** Ligand Interactions with UGMs

	Type of interaction	*Af*UGM_red_	*Ec*UGM_ox_	*Kp*UGM_red_	*Dr*UGM_red_
UDP-Gal*p* contacts	π-π stacking with uracil	Y104, F158	n.a.*	F152, Y155	F176, Y179
	H-bonding with uracil	F106, Q107		N270	F175, N296
	Interactions with diphosphate	R182, Y317 R327, Y419 Y453		R174, Y185, R280, Y314	R198, Y209 R305, Y335, Y370
	H-bonding with Gal*p*	R182, N207 N457		N84, Y349	H109, R305
	Other important amino acids for substrate binding	N163, W167		W160	T180, W184
FAD contacts	H-bonding with ribose	H63, G456, S461	N39, Y347	H60, L350, T355	H85 Y371
	Interaction with pyrophosphate	T18, L46 R447	F12, N39 R340	F13, S14, N41, R343	F39, A40 N67, R364
	π-π stacking with isoalloxazine	H63	H56	H60	H85
	Interaction with adenine	D38, S39, V242	E31, K32, D212,F213	F219	R60, D242 Y243
	H-bonding with isoalloxazine	V64, Q458	I57, M349, Y346	I61, M352	I86, M373
	H-bonding with N5	G62	A55	P59	P84
	H-bonding with ribose	D38	E31		D59
FAD-substrate contacts		OH-4(Gal*p*) and CO-4(FAD)	n.a.*	OH-4(Gal*p*) and CO-4(FAD)	OH-4(Gal*p*) and CO-4(FAD)

* Not available.

**Table 4.** Amino Acid Composition and Molecular Weight of UGMs from Different Organisms

Organism	Oligomeric state in solution	Number of amino acids	MW of monomer, Da	Ref.
*A. fumigatus*	Tetramer^a^,^b^	510	56,820	[59, 64]
*T. cruzi*	Monomer^a^	480	54,690	[73]
*L. major*	Monomer^a^	491	54,970	[96]
*E. coli*	Dimer^c^,^d^	367	42,970	[63, 97]
*K. pneumoniae*	Dimer^d^	384	44,460	[58, 61-62]
*M. tuberculosis*	Dimer^d^	399	45,820	[58]
*D. radiodurans*	Not determined	397	45,700	[56, 98]

a Determined by size exclusion chromatography.

b Determined by SAXS.

c Determined by light scattering.

d Inferred from analysis of protein-protein interfaces in the crystal lattice.

**Table 5.** Steady State Kinetic Parameters of UGMs from Different Organisms

Organism	k_cat_, s^-1^	K_M_, µM	k_cat_/K_M_, µM^-1^ s^-1^	Ref.
*A. fumigatus*	72 ± 4*a*	110 ± 15*a*	0.65 ± 0.09*a*	[59, 64]
*T. cruzi*	13.4 ± 0.3*a*; 11.5 ± 0.4*b*; 8.4 ± 0.9*c*	140 ± 10*a*; 200 ± 20*b*; 690 ± 150*c*	0.093 ± 0.006*a*; 0.056 ± 0.005*b*; 0.012 ± 0.001*c*	[73]
*L. major*	5 ± 0.2*a*	87 ± 11*a*	0.057 ± 0.006*a*	[96]
*E. coli*	27*a*	22*a*	1.22*a*	[99]
*K. pneumoniae*	5.5 ± 0.7*a*	43 ± 6*a*	0.12 ± 0.02*a*	[100]
*M. tuberculosis*	8	13	0.62	[101]
*D. radiodurans*	66 ± 2.4*a*	55 ± 7*a*	1.18*a*	[56]

a Reduced with 5-20 mM dithionite.

b Reduced with 0.5 mM NADPH.

c Reduced with 2.5 mM NADH.

**Table 6.** Kinetic Parameters of *Tc*UGM Reduction with NAD(P)H [73].

Substrate	*k*_red_, s^-1^	*K*_d_, µM	*k*_red_/*K*_d_, µM^-1^ s^-1^

NADH	0.085 ± 0.0006	550 ± 10	0.00015 ± 0.000002
NADPH	0.600 ± 0.006	98 ± 3	0.0061 ± 0.0001

## ABBREVIATIONS

UDP: = Uridine diphosphate
UGM: = UDP-galactopyranose mutase
Gal*p*: = Galactopyranose
Gal*f*: = Galactofuranose
WHO: = World Health Organization
NTD: = Neglected Tropical Diseases
*A. fumigatus*: = *Aspergillus fumigatus*
*A. niger*: = *Aspergillus niger*
*T. cruzi*: = *Trypanosoma cruzi*
*L. major*: = *Leishmania major*
*Leishmania spp.*: = *Leishmania species*
ABPA: = Allergic bronchopulmonary aspergillosis
IPA: = Invasive pulmonary aspergillosis
AIDS: = Acquired Immune Deficiency Syndrome
ICU: = Intensive care unit
GIPLs: = Glycoinositolphospholipids
GPI: = Glycosylphosphatidylinositol
FAD: = Flavin adenine dinucleotide
NAD(P)H: = Nicotinamide adenine dinucleotide (phosphate)
SAXS: = Small-angle X-ray scattering
LFER: = Linear free energy relationship
HTS: = High throughput screening
HPLC: = High performance liquid chromatography
UV/Vis: = Ultraviolet/visible
FP: = Fluorescence polarization
TAMRA: = Tetramethylrhodamine
